# Science Illustrated

**Published:** 1881-06

**Authors:** 


### SCIENCE ILLUSTRATED.

☞ The May number of the Scientific News, is at hand, with its very interesting and useful scientific information. Its illustrations, which are many, are striking and beautiful, conveying in most instances a definite and accurate impression at once, that would be with difficulty attained by word description. An example of this, is given on the first page, in the illustration of cut glass table-ware. The engravings are all good, and present their subjects fully.

The subjects presented in this work are such as are of every-day interest to almost everybody, and instructive, especially to the young. Familiarity with its contents will add to any and every one's stock of scientific knowledge, and impart a broader culture. It is published by Munn & Co., 37 Park Row, New York. Monthly, at $1.50 per year. *Send for it.*

We are indebted to Dr. W. Leigh Burton, of Richmond, Va., for copies of the Richmond "Baton." This little paper is edited by the Doctor, and is devoted to the interests of the Mozart Musical Association. Its columns are made up mostly of humorous items sparkling and witty, the reading of which brings about the merry grin that drives dull care away, and prolongs life—of course, Dr. B. being himself a member of the dental profession, does not neglect to glorify her in an occasional lay, as is shown by the following:

"My mother's teeth are going fast,
And ache from day to day;
At best, they'll not much longer last,
So swiftly they decay."

"She suffers now the direst pangs,
Which fall to human lot;
From aching teeth and broken fangs,
Which cease from troubling not."

"Ah! when she was a giddy girl,
And was a house-hold pet;
Each was then a faultless pearl,
In lips of ruby set."

"And now if she but has them drawn,
And gets a nice new set,
A new life will upon her dawn,
She may be happy yet."

---

Again:

"Father's teeth are plugged with zinc—
But let the truth be told;
The operator did not think,
That he would pay for gold."

"But as he did not have the cash
To pay for even lead;
I wish I'd given his nose a mash,
Or plugged his head instead."

In the graver walks of literature, Dr. Burton wields a pen fluent and forcible, and at the same time, with an elegance of diction, that is somewhat rare in these days of scribbling. It is to be regretted that the Doctor does not give the profession the benefit of an occasional effusion. We understand, however, that he is a gentleman, modest, retiring in disposition and withal, handsome; a rare combination, surely. This may explain the reason of his being so loth to present himself in letters to the profession.

IT APPEARS that the Cincinnati Lancet and Clinic, one of the liveliest and most enterprising of our medical journals does not intend that our daily papers shall monopolize telegraphic facilities for obtaining news, for in its last issue it contains a full telegraphic report of the proceedings of the American Medical Association, which it laid before its readers the day after the meeting adjourned. We believe, this is the first instant of telegraphic reporting by a medical journal.

